# Subcutaneous Emphysema, Pneumomediastinum, Pneumoretroperitoneum, and Pneumoscrotum: Unusual Complications of Acute Perforated Diverticulitis

**DOI:** 10.1155/2014/431563

**Published:** 2014-07-17

**Authors:** S. Fosi, V. Giuricin, V. Girardi, E. Di Caprera, E. Costanzo, R. Di Trapano, G. Simonetti

**Affiliations:** Department of Diagnostic Imaging, Molecular Imaging, Interventional Radiology and Radiation Therapy, University Hospital Tor Vergata, Viale Oxford 81, 00133 Rome, Italy

## Abstract

Pneumomediastinum, and subcutaneous emphysema usually result from spontaneous alveolar wall rupture and, far less commonly, from disruption of the upper airways or gastrointestinal tract. Subcutaneous neck emphysema, pneumomediastinum, and retropneumoperitoneum caused by nontraumatic perforations of the colon have been infrequently reported. The main symptoms of spontaneous subcutaneous emphysema are swelling and crepitus over the involved site; further clinical findings in case of subcutaneous cervical and mediastinal emphysema can be neck and chest pain and dyspnea. Radiological imaging plays an important role to achieve the correct diagnosis and extension of the disease. We present a quite rare case of spontaneous subcutaneous cervical emphysema, pneumomediastinum, and pneumoretroperitoneum due to perforation of an occult sigmoid diverticulum. Abdomen ultrasound, chest X-rays, and computer tomography (CT) were performed to evaluate the free gas extension and to identify potential sources of extravasating gas. Radiological diagnosis was confirmed by the subsequent surgical exploration.

## 1. Introduction

Subcutaneous cervical and mediastinal emphysema usually can occur as a result of surgery or trauma. Their spontaneous onset in absence of previous disorders or provocating factors is very rare.

Potential sources of extravasating gas are the respiratory tract (pneumothorax and bronchial fistula), the gastrointestinal tract (perforation), and infective causes (necrotising fasciitis) [1].

Pneumomediastinum and subcutaneous emphysema are very rare; reported signs of colonic perforation most often are associated with diverticulitis, toxic megacolon, and colonoscopy [2].

The continuum of fascial planes connecting cervical soft tissues with the mediastinum and retroperitoneum allows this dissection [3].

Clinical appearance depends on the degree and the extension of emphysema, and typical findings of spontaneous subcutaneous emphysema are swelling and crepitus over the involved site.

Imaging studies are helpful to confirm the diagnosis on doubtful cases, exclude local associated complications, determine the extension, and monitor the evolution.

Most commonly the diagnosis of subcutaneous emphysema and pneumomediastinum is made by chest X-ray, except in case of small gas collections that can be identified only by chest CT scan.

We describe a rare case of spontaneous subcutaneous emphysema, extending from the soft tissues of the abdominal wall to the neck, pneumomediastinum, and pneumoretroperitoneum resulting from an unknown sigmoid diverticulum perforation.

## 2. Case Presentation

A 57-year-old man was referred to our institution with a few days' history of severe left iliac fossa pain and pyrexia. The patient medical history included chronic diverticular disease.

At clinical examination he was pasty, sweaty, tachycardic and tachypneic, and in pain and he had a temperature of 38°C. The abdomen was slightly distended, vaguely tender on palpation over the lower region, without signs of peritoneal irritation, but he became febrile and confused. Peristalsis remained active and bowel function was normal.

His initial blood tests showed polycythemia and rise up transaminases.

The patient was transferred to our radiology department for ultrasonography exams, basic X-ray studies, and CT scanning.

We performed an abdomen ultrasonography that excluded subdiaphragmatic parenchymal tissue alterations.

Chest X-ray showed no sign of pneumothorax or pneumomediastinum.

**Figure 1:**
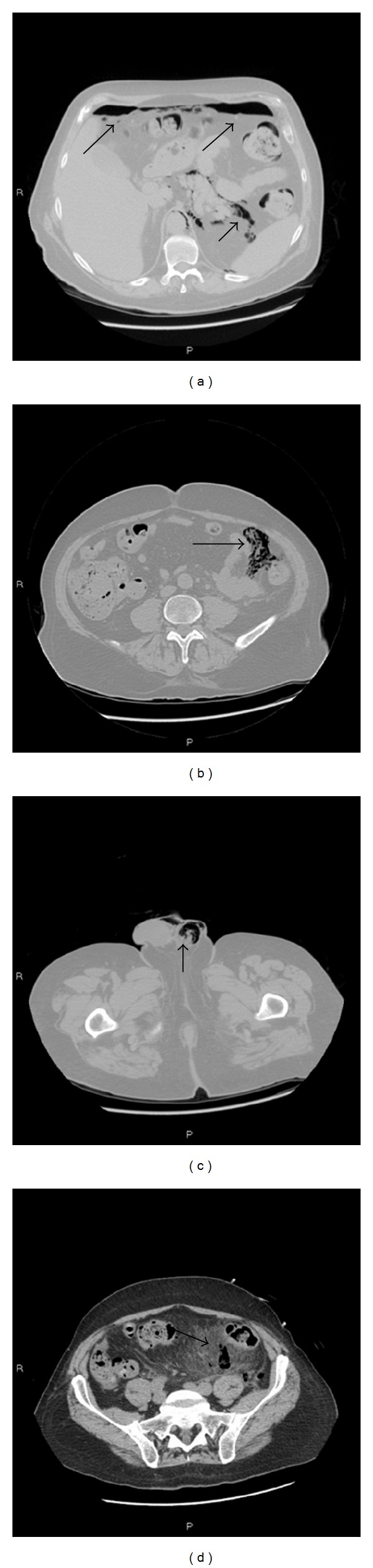
The first abdomen CT scan revealed free gas in correspondence of anterior subdiaphragmatic region (a) and left parietocolic shower (b). Gas was visualized within the scrotal sac (pneumoscrotum) (c). Peritoneal fat stranding and inflammatory changes are observed (d) (black arrows).

A CT scan of the abdomen (Figure 1) showed intra-abdominal free gas in correspondence of anterior subdiaphragmatic region, left anterior and posterior crural region, and ipsilateral parietocolic shower. Free gas was also noted in the retroperitoneum in correspondence of the proximal portion of the sigmoid colon.

Gas was finally visualized within the scrotal sac, indicative of pneumoscrotum.

CT scan revealed also mesenteric and mural thickening of the sigmoid colon, multiple sigmoid diverticula, peritoneal fat stranding, and evidence of inflammatory changes.

Considering patient's history and clinical and radiological features, the main diagnostic hypothesis was a diverticular perforation.

A few hours later the clinical evaluation showed subcutaneous emphysema and crepitus developed in correspondence to neck's soft tissue, to sternoclavicular joints, and to maxillary muscles bilaterally so a second chest radiograph was performed.

**Figure 2:**
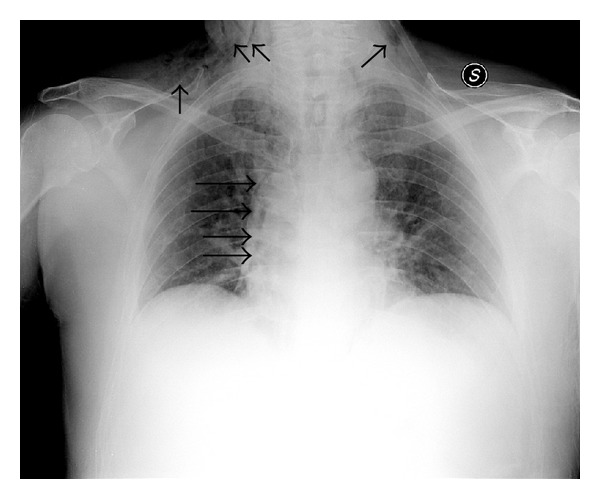
Chest X-ray showed subcutaneous emphysema, with gas tracking into the neck area bilaterally and a paracardiac gas stripe of the right (black arrows).

The radiograms (Figure 2) showed subcutaneous emphysema with gas tracking into the neck area bilaterally and a paracardiac gas stripe of the right that was suggestive for right-sided pneumomediastinum. There was no evidence of pneumothorax. The trachea was midline and the remainder of cardiac silhouette was normal.

Due to the clinical and radiological worsening, the patient underwent a new computed tomographic evaluation.

**Figure 3:**
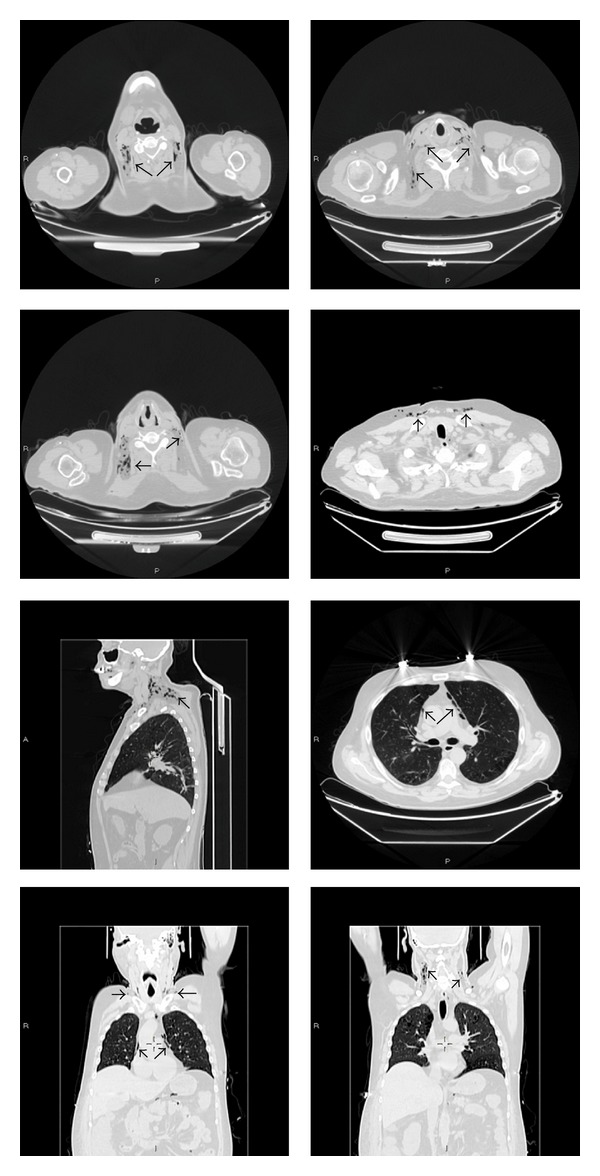
Whole body CT scan revealed free gas in the mediastinum and subcutaneous tissue, dissecting soft tissues and muscles from the maxillary to the sternocleidomastoid muscle and the sternoclavicular joint bilaterally. Free gas was also evident in correspondence of the dorsal and pectoral soft tissues (black arrows).

The whole body CT scan (Figure 3) revealed the onset of extraperitoneal free gas in the mediastinum and subcutaneous tissue that dissected soft tissues and muscles from the maxillary to the sternocleidomastoid muscle and the sternoclavicular joint bilaterally.

Free gas was also noted in correspondence of the dorsal and pectoral soft tissues.

This condition was suggestive of some communication between the chest and the retroperitoneum. Based on the CT scan, no other possible cause of the extraperitoneal gas except a diverticular perforation was found.

After the tomography, an urgent exploratory laparotomy was undertaken, under the suspicion of sigmoid diverticulitis rupture. During the operation, a perforation over the posterior wall of the sigmoid colon was found. Segmental resection of the sigmoid colon and end-colostomy (Hartmann's procedure) were performed.

Histologically, severe inflammatory changes with a mixed population of leucocytes, edema, and fresh haemorrhage at the edges of the rupture site were observed, with no sign of malignancy. The patient's subcutaneous emphysema and pneumomediastinum disappeared few days after the operation. One month later, the patient was still alive.

## 3. Discussion

Pneumomediastinum and subcutaneous emphysema are uncommon clinical entities that occur when gas leaks from the lungs or any of the luminal organs, such as the bronchial tube, larynx, trachea, esophagus, and very rarely, the colon, with subsequent dissection into the mediastinum [3].

They can occur in trauma: subcutaneous emphysema has been observed in hanged persons and reported in the literature.

In these cases, the mechanism of closing of the upper airways by compression of the larynx or pharynx and pressure of the tongue against the palate with an increase of intrathoracal pressure was assumed to be the main pathogenesis [4].

Another rare cause has been described in a case of sudden infant death syndrome with intravascular gas due to intraosseous medication application [5].

In this case gas embolism may passively occur when a puncture is made in a vessel with a pressure lower than the atmospheric one. The gas can enter the vessel when the needle is disconnected from the syringe to change medication or by accident [5].

Emesis may also cause esophageal rupture, which may lead to pneumomediastinum and subcutaneous emphysema [6].

Instead spontaneous pneumomediastinum commonly occurs when an increased intra-alveolar pressure (asthma, cough) leads to the rupture of the marginal pulmonary alveoli. The air ascends along the bronchi to the mediastinum and the subcutaneous space of the neck, causing cervicofacial subcutaneous emphysema in 70–90% of cases [4].

In the literature there are only a few cases of subcutaneous emphysema of gastrointestinal origin reported [7–9], that usually occurs after surgical procedures, especially in case of leakage of suture lines, fistula formation, or infections.

Other causes include Borehaave's syndrome, perforated peptic ulcers, traumatic perforation, appendicitis, and diverticulitis [9].

The anatomical site of perforation largely determines the route of escape of the gas to the subcutaneous position. In addition, the direction of gas diffusion usually follows the least resistance, loose areolar fascial structures [10].

In our case the perforation was located in the posterior wall of the sigmoid colon, so the escaped gas penetrated first into the retroperitoneal space and then may continue diffusing superiorly through the paravertebral retroperitoneal tissues and diaphragmatic hiatus into the mediastinum and finally into the neck and facial areas.

Diagnosis is made most commonly by chest X-ray, which reveals subcutaneous emphysema and pneumomediastinum. Small pneumomediastinum not seen on chest X-ray will be seen on chest CT.

Although, as mentioned above, subcutaneous neck emphysema, pneumomediastinum, and retropneumoperitoneum are rarely caused by nontraumatic perforations of the colon, this possibility should be considered when no obvious case can be found for the origin of free gas at these sites.

In this case, abdomen CT scan plays an important role in identifying an occult perforation and whole body CT allows us to evaluate the extension of escaped gas to different body districts.

In fact, in cases of suspected soft tissues emphysema, for a fast management of the patient, we recommend performing a CT, that is, the most preferable diagnostic tool for the detection of air in soft tissues [4].

Finally radiological imaging plays also an important role in allowing a proper therapeutic planning and followup.

## 4. Conclusion

We conclude that performing CT is very useful in making a secure diagnosis of pneumomediastinum and soft tissue emphysema because it allows us to define a precise extension of gas to different body districts.

Patients can be scanned very quickly and easily and other information supplied by CT is also necessary to achieve the final diagnosis and ruling out other causes of pneumomediastinum and soft tissues emphysema.

Radiological evidence of pneumomediastinum and soft tissues emphysema may lead to early diagnosis and surgical management with a better outcome.
